# A pre-formulation study of tetracaine loaded in optimized nanostructured lipid carriers

**DOI:** 10.1038/s41598-021-99743-6

**Published:** 2021-11-02

**Authors:** Simone R. Castro, Lígia N. M. Ribeiro, Márcia C. Breitkreitz, Viviane A. Guilherme, Gustavo H. Rodrigues da Silva, Hery Mitsutake, Ana C. S. Alcântara, Fabiano Yokaichiya, Margareth K. K. D. Franco, Daniel Clemens, Ben Kent, Marcelo Lancellotti, Daniele R. de Araújo, Eneida de Paula

**Affiliations:** 1 Department of Biochemistry and Tissue Biology, Institute of Biology, University of Campinas (UNICAMP), Rua Monteiro Lobato 255, Campinas, SP 13083862 Brazil; 2 Institute of Biotechnology, Federal University of Uberlandia, Uberlandia, MG Brazil; 3 Department of Analytical Chemistry, Institute of Chemistry, UNICAMP, Campinas, SP Brazil; 4 Department of Chemistry, Federal University of Maranhão, São Luís, MA Brazil; 5 Department of Quantum Phenomena in Novel Materials, Helmholtz-Zentrum, Berlin, Germany; 6 Nuclear and Energy Research Institute, IPEN–CNEN/SP, São Paulo, SP Brazil; 7 Faculty of Pharmaceutical Sciences, UNICAMP, Campinas, SP Brazil; 8 Human and Natural Science Center, Federal University of ABC (UFABC), Santo André, SP Brazil

**Keywords:** Supramolecular assembly, Nanostructures

## Abstract

Tetracaine (TTC) is a local anesthetic broadly used for topical and spinal blockade, despite its systemic toxicity. Encapsulation in nanostructured lipid carriers (NLC) may prolong TTC delivery at the site of injection, reducing such toxicity. This work reports the development of NLC loading 4% TTC. Structural properties and encapsulation efficiency (%EE > 63%) guided the selection of three pre-formulations of different lipid composition, through a 2^3^ factorial design of experiments (DOE). DLS and TEM analyses revealed average sizes (193–220 nm), polydispersity (< 0.2), zeta potential |− 21.8 to − 30.1 mV| and spherical shape of the nanoparticles, while FTIR-ATR, NTA, DSC, XRD and SANS provided details on their structure and physicochemical stability over time. Interestingly, one optimized pre-formulation (CP-TRANS/TTC) showed phase-separation after 4 months, as predicted by Raman imaging that detected lack of miscibility between its solid (cetyl palmitate) and liquid (Transcutol) lipids. SANS analyses identified lamellar arrangements inside such nanoparticles, the thickness of the lamellae been decreased by TTC. As a result of this combined approach (DOE and biophysical techniques) two optimized pre-formulations were rationally selected, both with great potential as drug delivery systems, extending the release of the anesthetic (> 48 h) and reducing TTC cytotoxicity against Balb/c 3T3 cells.

## Introduction

Local anesthetics prevent pain by interrupting nerve excitation and conduction. They interact with voltage-gated sodium channels, blocking the propagation of nerve impulses^1,2^. Tetracaine (TTC) is a potent anesthetic of the amino ester class, which fast metabolism by plasma esterases and high systemic toxicity^3^ have restricted its use to topical anesthesia in ophthalmic procedures, oropharyngeal pain, skin anesthesia and spinal block^2,4,5^.

In order to enhance the therapeutic effect and reduce the toxicity of local anesthetics, drug delivery systems (DDS) have shown promising results, in nanocarriers such as liposomes, cyclodextrins, polymeric particles^6^, solid lipid nanoparticles^7,8^, and nanostructured lipid carriers (NLC)^9,10^. NLC are colloidal systems, which consist of a lipid matrix composed of a mixture of solid and liquid (oils) lipids, plus a surfactant. The lipid matrix is a pseudo-crystalline structure with many imperfections^11^ that confer superior physicochemical stability to the nanoparticle and allow drug insertion, increasing its solubility, permeability, bioavailability and reducing adverse effects^12^. In comparison to other lipid-based carriers (e.g. liposomes, solid lipid nanoparticles), NLC have longer shelf stability and higher upload capacity for lipophilic drugs^10^.

Here we report the development, among different lipid matrices, of novel DDS for tetracaine. Factorial design was used as a chemometric tool to elucidate the effect of excipients in the NLC supramolecular organization, providing robust systems with optimized structural properties for subsequent analyses^13^. Among other biophysical techniques, Raman imaging and SANS analyses disclosed details on the lipid matrix organization, that reduced the shelf stability of one optimized formulation. Two pre-formulations were then selected, aiming at improving TTC local anesthetic action (CP-DK/TTC and MM-CK/TTC) for further application in clinics. They showed excellent shelf-stability (> 1 year), high upload capacity (> 60%), prolonged drug release (> 48 h) and reduced cytotoxicity against Balb/c 3T3 fibroblasts.

## Materials and methods

Tetracaine base (TTC) was purchased from AK Scientific, Inc. (Union City, USA). The surfactants Pluronic F68 (P68) and Tween 80 were supplied by Sigma (St. Louis, USA); cetyl palmitate (CP) and Dhaykol 6040 (DK)—or capric/caprylic triglycerides—by Dhaymers Quím. Fina (Taboão da Serra, Brazil); myristyl myristate (MM) by Croda (Campinas, Brazil) and diethylene glycol monoethyl ether or Transcutol (TRANS) by Gattefossé (Lyon, France). HPLC-grade acetonitrile and methanol were purchased from J.T. Baker (Allentown, USA). 3T3 cells were from American type culture collection (ATCC, Manassas, VA, USA). 3-(4,5-dimethylthiazol-2-yl)-2,5-diphenyltetrazolium bromide (MTT), RPMI medium, fetal bovine serum, penicillin, streptomycin and uranyl acetate were purchased from Sigma-Aldrich (Missouri, USA). All other reagents were of analytical grade. Deionized water (18 MΩ) was obtained from an Elga USF Maxima ultra-pure Water purifier.

### NLC preparation

NLC of different matrices (CP-TRANS, CP-DK and MM-DK) were obtained by emulsification-ultrasonication^14^. The oily phase was prepared by melting solid and liquid lipids in a water bath, 10 °C above the melting temperatures of the solid lipid (42.6 and 55.0 °C for MM and CP, respectively)^8,15^. TTC was added to the mixture of melted lipids and stirred until complete solubilization. Simultaneously, an aqueous phase was prepared by dissolving the surfactant (P68) in heated deionized water. The aqueous phase was then poured into the lipid phase under stirring at 10,000 rpm (Ultra-Turrax T18, IKA T18 basic) for 3 min. The obtained microemulsion was sonicated in a tip sonicator (Vibracell, Sonics & Materials Inc.) at 500 W and 20 kHz for 30 min, in alternating on/off 30 s cycles. The resulting nanoemulsion was immediately cooled to room temperature in an ice bath to produce the NLC.

### Experimental design

Three 2^3^ factorial designs were carried out, with triplicates at the central point, for each of the three lipid combinations: CP-TRANS, CP-DK and MM-DK. Experimental data were processed using Design-Expert software (version 9.0, State-Ease Inc., Minneapolis, USA). Analysis of variance (ANOVA) was applied to evaluate the significance of the effects, their interactions, and the lack of adjustment of the regression model, considering the confidence level of 95% (α = 0.05). Three independent variables were used: (a) surfactant concentration (% P68), (b) total lipid concentration (% TL), and (c) solid:liquid lipid ratio (expressed as % SL), eleven NLC formulations (of nine different compositions) containing 4% TTC were prepared in each of the 2^3^ factorial design. Table 1 shows the variables and levels used in this study, for each lipid combination.

**Table 1:** Experimental variables and their levels, responses and optimization goals for the 2^3^ experimental designs of CP-TRANS, CP-DK and MM-DK nanostructured lipid carriers.

Independent variables	Levels		
	− 1	0	+ 1
Surfactant concentration (% P68 w/v)	2	3.5	5
Lipid phase concentration (% TL w/v)	16	18	20
Solid:liquid lipid ratio (% SL:LL w/w)	70:30	80:20	90:10

Dependent variables		Goal	
Particle size (nm)		Minimum	
Polydispersity index (PDI)		Minimum	
Zeta potential (|mV|)		Maximum	

The composition of the eleven formulations prepared according to the factorial design are shown in Table S1. They were all prepared in a random order and the central point triplicates encompassed all the sample preparation steps (authentic replicates).

### Determination of particle size, polydispersity, zeta potential, concentration and pH

The particle size (hydrodynamic diameter) and polydispersity index (PDI) were determined by dynamic light scattering (DLS) and zeta potential (ZP) by electrophoretic mobility, in a Nano ZS90 analyzer (Malvern Instruments, UK), at 25 °C. The samples were diluted (1000×) in deionized water. The pH of NLC formulations were measured with a R-TEC7 pH meter (Tecnal Equip. Cient., Piracicaba, Brazil), at 25 °C (n = 3).

The concentration of nanoparticles in the formulations was determined by Nanotracking analysis (NTA) in a NS300 (NanoSight, Amesbury, UK) equipment. The samples were diluted in deionized water (5000×) and injected into the sample chamber with syringes. All measurements were performed at 25 °C, (n = 3).

### Quantification of tetracaine by HPLC

TTC quantification, in triplicate, was performed in a Varian ProStar high-performance liquid chromatograph (HPLC) equipped with a PS 325 UV–Vis detector, PS 210 solvent delivery module and Galaxy Workstation software for data collection. A Luna (5 μ, 250 × 4.6 mm) reverse-phase C18 column (Phenomenex, Torrance, US) was used. Isocratic elution was run with a mobile phase of ammonium phosphate (10 mM, pH 3.0): acetonitrile, 70:30 v/v. The flow rate, injection volume and wavelength detection were set at 1.5 mL/min, 30 µL and 280 nm, respectively. TTC retention time was = 5.3 ± 0.1 min. The assay method was validated, and the calibration curve was linear (r^2^ = 0.9998) within the concentration range of 0.03–1.2 mM. The limits of detection and quantification of TTC were 4.47 × 10^–5^ mM and 1.49 × 10^–4^ mM, respectively.

### Encapsulation efficiency and drug loading determination

TTC encapsulation by the three NLC formulations (CP-TRANS/TTC, CP-DK/TTC and MM-DK/TTC) was determined in triplicate, by centrifugation-ultrafiltration, using 10 kDa pore size Amicon regenerated cellulose filters (Millipore Corp., Bedford, USA). The samples were diluted in deionized water and centrifuged at 6000×*g* for 20 min. The filtered aqueous solution was collected and free tetracaine was quantified at 280 nm, by HPLC. The amount of TTC loaded in the NLC was expressed in terms of encapsulation efficiency (%EE), calculated according to Eq. (1), or drug loading capacity (%DL), accordingly to Eq. (2)^16^:

1 $$\% {\text{EE}} = \frac{{TTC_{total} - TTC_{free} }}{{TTC_{total} }} \times 100$$

2 $$\% \;Drug\;loading = \frac{weight\;of\;encapsulated\;TTC}{{weight\;of\;nanoparticles}} \times 100$$

### Transmission electron microscopy (TEM) images

The morphological analysis of the nanoparticles in all the three systems and their respective controls (prepared without TTC) was performed by transmission electron microscopy. 2% Uranyl acetate was added to the diluted samples, for contrast. Aliquots of the samples were then deposited on copper grids coated with carbon film and dried at room temperature. After drying, micrographs of the samples were obtained in a JEOL 1200 EXII microscope, at 60 kV and the images were edited with ImageJ software v.1.52a (https://imagej.nih.gov/ij/).

### Infrared spectroscopy measurements (ATR-FTIR)

ATR-FTIR analyses were performed for excipients, lyophilized formulations containing 4% TTC (CP-TRANS/TTC, CP-DK/TTC and MM-DK/TTC) and their respective controls (CP-TRANS, CP-DK, MM-DK) without the anesthetic. Spectra were recorded in FTIR spectrophotometers (Bruker IFS 66 v/S or Perkin Elmer Spectrum 65) equipped with ATR cells, in the reflection mode, between 4500 and 500 cm^−1^.

### Differential scanning calorimetry (DSC) analysis

DSC measurements of TTC, lyophilized NLC formulations (CP-TRANS/TTC, CP-DK/TTC, MM-DK/TTC), their controls (CP-TRANS, CP-DK, MM-DK), and solid lipid excipients (CP, MM) were taken in a TA Q20 calorimeter (Thermal Analysis Instruments, New Castle, USA) equipped with a cooling system. The samples (5 mg) were placed in aluminum pans and the thermal profiles were obtained in the range of 0–250 °C, at a heating rate of 10 °C/min, under nitrogen flow.

### X-ray diffraction (XRD) analysis

X-ray powder diffraction (XRD) data were obtained in a Shimadzu XRD7000 diffractometer (Tokyo, Japan), using a Cu-Kα source at a scanning step of 2° min^−1^, between values of 2θ (5°–50°). Samples of lyophilized NLC containing 4% TTC (CP-TRANS/TTC, CP-DK/TTC, MM-DK/TTC), their controls (CP-TRANS, CP-DK, MM-DK), and solid lipid excipients (CP, MM) were analyzed.

### Small Angle Neutron Scattering (SANS) analysis

SANS measurements were performed at the VSANS-V16 (time of flight/very small angle scattering) instrument at Helmholtz-Zentrum Berlin (Germany). The scattering data was gathered at two sample detector distances: 1.7 m with neutron wavelengths of 1.8–3.8 Å, and 11 m with 1.6–9.2 Å neutron wavelengths. The samples were placed in cuvettes (Hellma 110 QS) of 1 mm light path arranged in a sample holder that provides twenty positions per measurement. A waiting time of 30 min between measurements ensured the stabilization of the sample’s temperature. Samples of NLC formulations containing 4% tetracaine (CP-TRANS/TTC, CP-DK/TTC, MM-DK/TTC) and their controls (CP-TRANS, CP-DK, MM-DK), were prepared in D_2_O, and measured at 25 and 37 °C. The final data reduction included corrections for sample transmission, background detector counts, empty cell scattering and detector efficiency, and they were scaled to absolute intensity using a 1 mm H_2_O standard measurement. The SANS data were radially averaged and combined to give a total *q* range of 0.005–0.5 Å^−1^ ^17^. To gain a further insight about the nanoparticles containing DK (for which hydrophobic clusters between SL–LL were observed), their SANS data were modelled using the empirical function shown in Eq. (3):

3 $$I(q) = \frac{A}{{q^{n} }} + \frac{B}{{1 + (q\xi )^{m} }} + back$$

where A and B are constants, n and m are power-law indices, *back* refers to the incoherent background and ξ is the correlation length^17–24^. The first term in the equation is a power law decay (Porod-like scattering) which describes the scattering from clusters or aggregates in the system. The second term is a Lorentzian function that corresponds to the scattering of individual chains in solution (association contribution), where ξ is the key parameter.

### Physicochemical stability study

The physicochemical stability of the NLC formulations was monitored (n = 3) for 12 months at 25 ± 2 °C and 60 ± 5% relative humidity (ICH 2009). The evaluated parameters were nanoparticle size (nm), PDI and ZP (mV), plus visual analysis. Analysis of variance (ANOVA, p < 0.05) and Tukey’s post-hoc tests were used to compare significant differences over time.

### Lipid miscibility assessed by Raman mapping

The samples were prepared by heating the solid lipids (CP, MM) 10 °C above their melting temperatures, followed by addition of the liquid lipid (TRANS, DK) under stirring until a visually homogeneous mixture was obtained. The concentration of the liquid lipid was set to 30% (w/w) for the analyses. The samples were cooled to room temperature in an aluminum cell and an area of 1.95 × 1.95 mm^2^ was mapped in a Raman Station 400 (Perkin Elmer, Waltham, USA) using a laser of 785 nm as an excitation light and nominal power of 100 mW. The exposure time was set at 3 s/pixel, 2 exposures/pixel, 50 µm pixel size, in the spectral range of 3200–600 cm^−1^, with 4 cm^−1^ resolution. Each sample provided a data cube whose dimension was 40 × 40 × 651, where 40 represents the number of pixels at *x* and *y* axis, and 651 is the number of spectral variable/Raman shift.

### Chemometric analysis

Spikes on Raman spectra were excluded using an algorithm written in Matlab^25^. After exclusion of spikes, the data cube was unfolded to a 2D matrix, where pixel position on axis *x* and *y* were rows, and spectral variables were the columns. Thus, each sample provided a matrix of 1600 spectra × 651 variables (Raman shifts). The spectra were smoothed using Savitzky-Golay (width = 5, order of polynomial = 2), with baseline correction by weighted least squares and normalization by unit vector. The spectral range was 1804–724 cm^−1^ for CP-TRANS and MM-DK, and 2964–844 cm^−1^ for CP-DK samples. The classical least squares (CLS) method was used to generate the maps of concentration (chemical images). CLS considers that the spectrum of a mixture is the sum of the spectra of the pure compounds weighted by their concentrations^16,26^. The standard deviation of the histograms (SD_hist_) of these maps was used to assess the miscibility between SL and LL. Data analyses were performed using Matlab R2013b (Mathworks Inc., Natick MA, US) and PLS Toolbox version 8.2 (Eigenvector Research Inc., Wenatchee WA, US).

### In vitro release experiments

The in vitro release of TTC, free (in solution) or encapsulated by NLC (CP-TRANS/TTC, CP-DK/TTC, MM-DK/TTC), was measured using a Franz vertical diffusion cell system under sink condition^27^. The test samples (0.4 mL) were placed in the donor compartment of the diffusion cells, which was separated by a polycarbonate membrane (Nucleopore Track-Etch, 0.1 mm pore size, Whatman)^28^ from the acceptor compartment containing 4 mL of the release medium (5 mM PBS, pH 7.4 with 5% Tween 80). The system was kept at 37 °C under magnetic stirring (300 rpm). At predetermined intervals during 50 h, aliquots (0.2 mL) were extracted from the acceptor compartment and the volume was replaced with the release medium. The concentration of released TTC in the aliquots was determined by HPLC (n = 5).

The release curves were analyzed with the KinetDS 3.0 software^29^. Several kinetic models were tested and according to the coefficient of determination (R^2^) the best fit for NLC formulation curves was reached with the Korsmeyer–Peppas model:

4 $$Q = k \cdot t^{n}$$

where *Q* is fraction of drug released at time *t*, *k* is the rate constant, and *n* is the release exponent that typifies the drug release mechanism: *n* = 0.43 indicates Fickian diffusion, *n* = 1 means zero-order release while 0.43 < *n* < 1 values are related to anomalous transport^30^.

### Cell viability tests

Balb/c 3T3 murine fibroblasts were used and cell viability was measured by reduction of MTT. Cells were cultured in RPMI 1640 medium supplemented with 10% fetal bovine serum and 1% antibiotic (penicillin and streptomycin). Cells (1.0 × 10^4^ cells/mL^−1^) in RPMI medium and incubated in 96-well plates for 24 h at 37 °C under humidified atmosphere and 5% CO_2_ were treated with different concentrations of the samples (free TTC, CP-TRANS/TTC, CP-DK/TTC, MM-DK/TTC) diluted in the medium, for 24 h. The treatment medium was removed, the plates washed with sterile PBS buffer pH 7.4, and 100 μL of MTT solution (0.5 mg mL^−1^ in culture medium) was added to each well. After incubation for 3 h at 37 °C, the MTT solution was removed, and the formed formazan crystals were solubilized in 100 μL of ethanol. The plates were shaken for 5 min, and the absorbance of each well was read at 570 nm. Values were expressed as percent MTT reduction, in comparison to control (untreated cells). The analyses were performed in triplicate, and the results expressed as mean ± SD. The statistical analysis of the results was performed by One-way ANOVA, followed by Tukey’s test (p < 0.05).

## Results and discussion

This study reports the development of three optimized NLC pre-formulations capable of encapsulating TTC at high doses (4%). The NLC were prepared with mixtures of solid and liquid lipids (CP-TRANS, CP-DK and MM-DK), plus P68 as a stabilizer. The choice of lipids and their concentrations was based on previous reports^8,16^. Differently than a previous report in the literature in which TTC was incorporated in NLC aimed for topical application^31^ the formulation in here is to be used by different routes of administration.

### Experimental design

In order to analyze the factors that influence the different NLC formulations, three 2^3^ factorial designs with triplicate at the central point were carried out. Using three independent variables: (a) surfactant concentration (% P68), (b) total lipid concentration (% TL), and (c) solid:liquid lipid ratio (expressed as % SL), eleven NLC formulations (of nine different compositions) containing 4% TTC were prepared in each of the 2^3^ factorial design. Table S1 shows the composition of these systems and corresponding experimental responses (size, PDI, ZP), determined by DLS.

**Figure 1:**
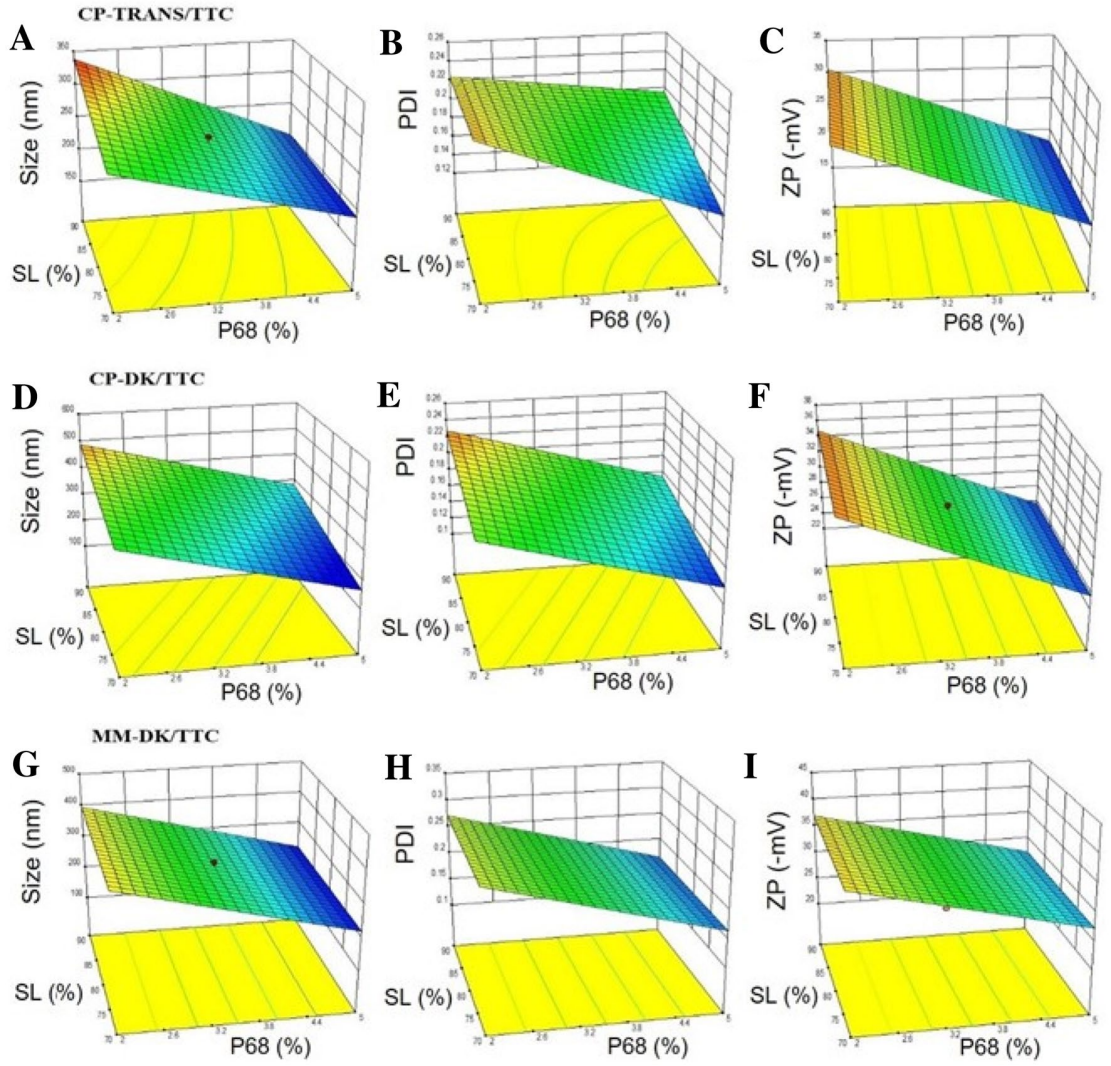
Response surfaces of the three different NLC formulations (CP-TRANS/TTC, CP-DK/TTC, MM-DK/TTC), at 18% total lipid concentration, regarding: size (**A**, **D**, **G**), PDI (**B**, **E**, **H**), and ZP (**C**, **F**, **I**).

The mean particle size of the NLC ranged from 250 to 330 nm. Table S2 shows that in the formulations composed of CP-TRANS/TTC, two variables significantly affected the particle size: % P68, % SL and their interactions (p < 0.05), as exemplified in Fig. 1A. For CP-DK/TTC and MM-DK/TTC only % P68 (Fig. 1D,G and Table S2) significantly influenced particle size. The positive effect of the amount of solid lipid (% SL) on the size of CP-TRANS/TTC nanoparticles is a consequence of the higher viscosity of the lipid phase, reducing the effectiveness of particle breaking (homogenization and sonication) processes^32,33^ and will also be discussed further (SANS and Raman Imaging data). For all the 3 NLC types the surfactant concentration (% P68) had a negative effect on the particle size, which means that the increase in the concentration of P68 decreases the particle size, due to the decrease of interfacial tension between the nanoparticles and the external phase^33^.

The low PDI values (0.1 > PDI > 0.25) in Table S1 confirmed the homogenous distribution of particle sizes for CP-TRANS/TTC, CP-DK/TTC and MM-DK/TTC. Figure 1B,E,H and Table S2 revealed that only P68 had a significant (negative) effect, decreasing PDI in the 3 NLC formulations, a result that confirms that surfactants play a major role in determining the size distribution of the nanoparticles.

ZP values in the three types of formulations were in the range of − 20 to − 40 mV (Table S1) and P68 was the only variable that significantly affected ZP values in a negative way: the higher the P68 concentration the lower, in modulus, the surface electric potential of the NLC (Table S2, Fig. 1C,F,I). The negative ZP values are attributable to the polarization of surfactant P68, followed by adsorption of water molecules on the polarized surfaces of NLC^32–37^.

Desirability functions were used to determine the preferred formulation, following the criteria: lower particle sizes, minimum PDI and maximum ZP values. According to that, the following systems were selected: CP-TRANS/TTC = 20% TL, 70:30% SL, and 4.4% P68, CP-DK/TTC = 18% TL, 70:30% SL, and 4.4% P68 and MM-DK/TTC = 17% TL, 72:28% SL, and 5% P68. The subsequent experiments were conducted only with these optimized formulations.

### DLS and NTA results

Table 2 shows the average size, PDI, ZP and nanoparticle concentration (NC) values for the optimized formulations revealed by Factorial Design, and their respective controls. The three types of NLC formulations displayed sub-micron diameters (~ 200 nm) and monodisperse size distribution (0.1 < PDI < 0.20) with proper electrical charge repulsion between the particles (ZP > |17| mV) to ensure good shelf-stability^36–40^. Visual analysis confirmed the homogeneous appearance of the fresh samples, with suspensions of whitish coloring, liquid consistency and no evidence of aggregates.

**Table 2:** Particle size, polydispersity (PDI), zeta potential (ZP), nanoparticle concentration (NC), encapsulation efficiency (%EE) and drug loading capacity (%DL) of the optimized NLC formulations (mean ± SD, n = 3).

Formulation	Size (nm)	PDI	ZP (mV)	NC (× 10^13^/mL)		%EE	%DL
CP-TRANS	166.6 ± 1.4	0.115 ± 0.031	− 17.4 ± 0.6		7.81 ± 0.43	–	
CP-TRANS/TTC	199.0 ± 2.5	0.187 ± 0.028	− 21.8 ± 1.5		7.77 ± 0.19	68.1 ± 3.5	11.1 ± 0.2
CP-DK	189.9 ± 2.9	0.112 ± 0.015	− 19.0 ± 0.9		11.50 ± 0.34	–	
CP-DK/TTC	215.8 ± 1.8	0.172 ± 0.010	− 27.8 ± 0.5		9.20 ± 0.98	65.0 ± 2.5	11.6 ± 0.4
MM-DK	193.3 ± 2.7	0.139 ± 0.021	− 29.0 ± 1.7		10.80 ± 0.33	–	
MM-DK/TTC	222.2 ± 2.6	0.154 ± 0.020	− 30.1 ± 0.3		6.77 ± 0.48	63.7 ± 4.2	11.6 ± 0.8

NTA is an alternative methodology to DLS for in vitro characterization of nanostructured colloidal systems^9^. NTA measurements allowed determination of the nanoparticles concentration (NC) in the optimized formulations (Table 2), an analytical parameter used in nanotoxicity, pharmacokinetic and stability (rupture, aggregation) studies of nanoparticles^41,42^. The slightly lower NC values of CP-TRANS/TTC, CP-DK/TTC, MM-DK/TTC reflect their higher sizes, in comparison to the controls without tetracaine.

Most importantly, we have used the NC values and %EE to estimate the number of molecules of each excipient per nanoparticle^8^, as shown in Table S3. These numbers revealed a significant number of TTC molecules inside each particle (6–8 × 10^5^), corresponding to TTC:total lipid molar ratios of 0.14–0.17 that justify the increased diameter of TTC-containing nanoparticles.

### Tetracaine encapsulation efficiency and drug loading

The three types of NLC formulations developed in this study showed very good capacity to carry TTC, with %EE values ranging from 63.7 to 68.1% and %DL > 11 (Table 2), reflecting the strong partition of the non-ionized form of TTC in the lipid milieu^43^. Indeed, the encapsulation efficiency determined for TTC in these nanoparticles was in the range of those reported (> 55%) for other hydrophobic local anesthetics such as dibucaine, bupivacaine and ropivacaine^16,44,45^ and higher (< 37%) than those observed in NLC with more hydrophilic agents such as lidocaine and prilocaine^8^. The drug loading capacity is another parameter that expresses the upload capacity of DDS (Eq. 2). The DL values of the optimized formulations for TTC were 11.1% with CP-TRANS/TTC and 11.6% with CP-DK/TTC and MM-DK/TTC, above those reported (%DL < 10) for other local anesthetics in NLC^16,46,47^. %DL values were also in good agreement with the TTC:TL ratios (Table S3).

### Transmission electron microscopy

**Figure 2:**
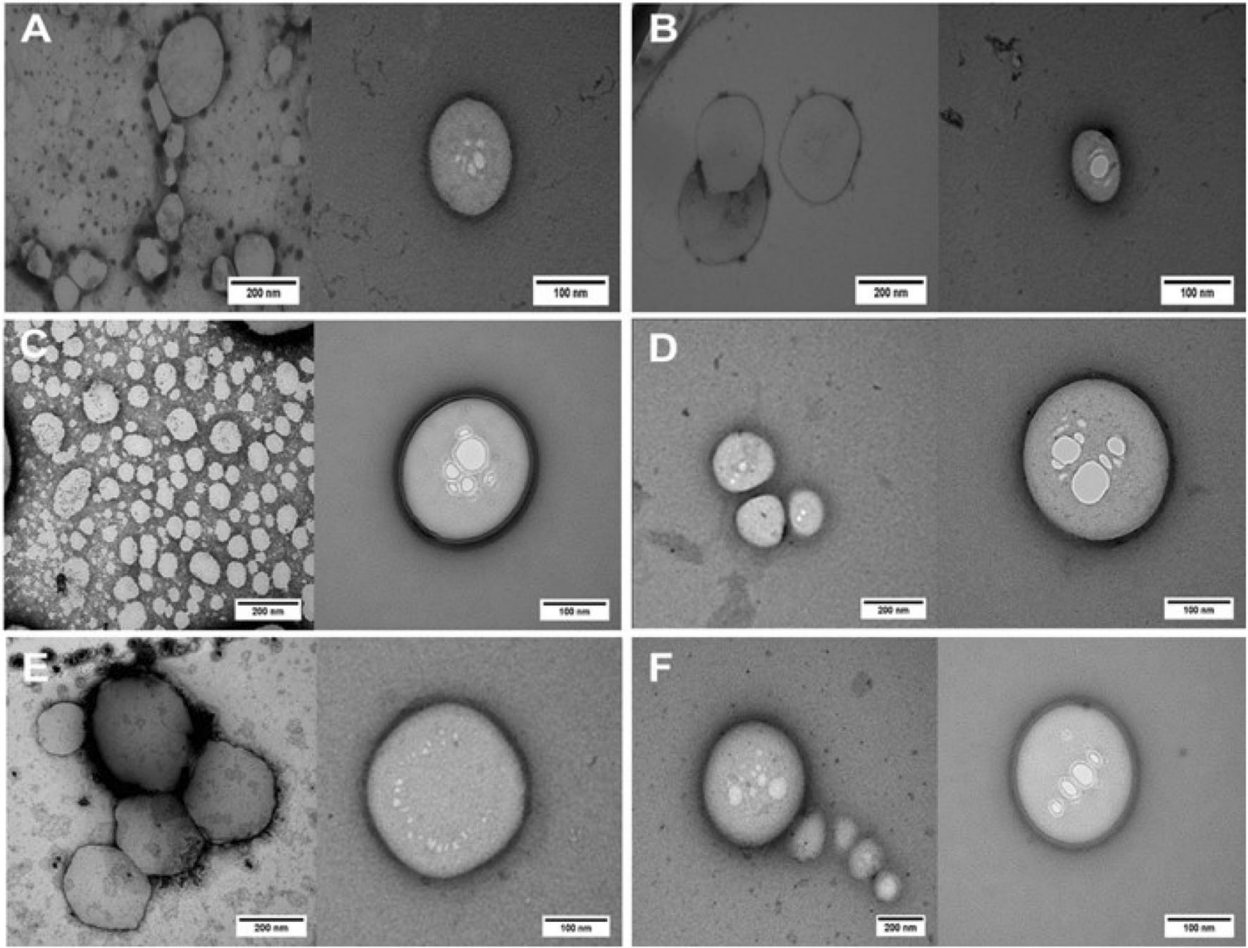
TEM images of NLC formulations and their controls (without TTC): CP-TRANS/TTC (**A**), CP-TRANS (**B**), CP-DK/TTC (**C**), CP-DK (**D**), MM-DK/TTC (**E**) and MM-DK. Magnification: ×60,000 (left) (**E**) ×100.000 (right). Images edited with ImageJ software v.1.52a (https://imagej.nih.gov/ij/).

TEM images showed that the nanoparticles, in despite of the different lipid matrices, had spherical morphology with a well-delimited surface (Fig. 2). In addition, encapsulation of TTC did not affect the integrity of the nanoparticles (Fig. 2A,C,E vs. B,D,F).

### Infrared analysis (ATR-FTIR)

**Figure 3:**
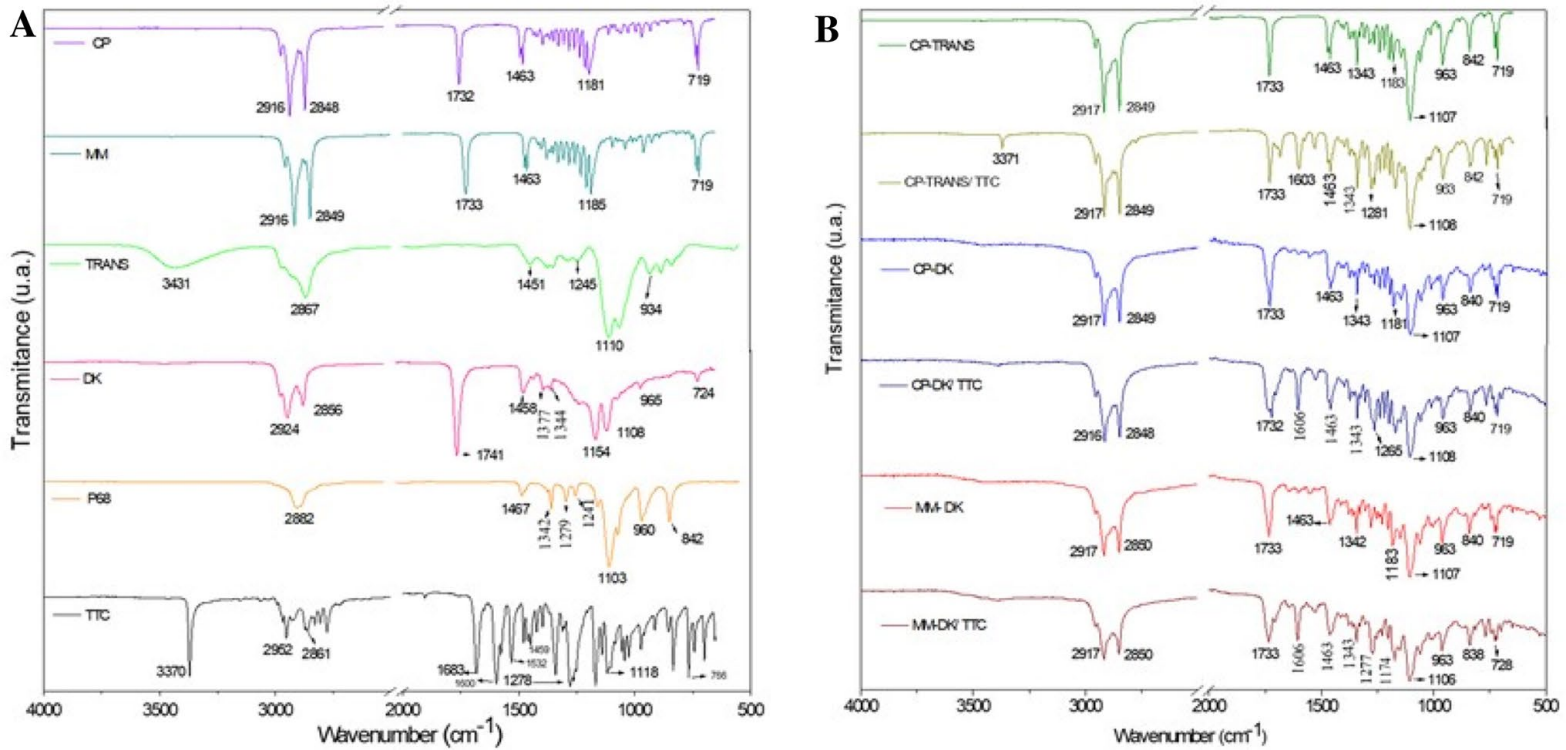
FTIR analysis of TTC, excipients (cetyl palmitate (CP), myristyl myristate (MM), Transcutol (TRANS), Dhaykol (DK), Pluronic F68 (P68), TTC-containing nanoparticles (CP-TRANS/TTC, CP-DK/TTC and MM-DK/TTC) and their controls (CP-TRANS, CP-DK, MM-DK).

ATR-FTIR analyses were performed to investigate possible interactions between the anesthetic and the lipid matrix of NLC. Figure 3A shows the spectra of NLC excipients and TTC, while the spectra of the optimized NLC formulations and their controls are given in Fig. 3B. TTC spectrum showed absorption bands at 3370 and 1532 cm^−1^ attributed to N–H groups and C–N stretching vibrations from the aromatic amine group, respectively, while the bands at 2952 and 2861 cm^−1^ are due to asymmetric CH_3_ stretching and symmetrical CH_2_ stretching vibrations, respectively. Other bands at 1683 cm^−1^ corresponding to the C=O stretching vibration of ester and 1600 cm^−1^ due to C=C of the aromatic ring were detected, as well as those at 1168 and 1118 cm^−1^ which refer to antisymmetric and symmetric stretching of C–O–C, respectively^5,48^.

Control NLC (CP-TRANS, CP-DK and MM-DK) showed bands related to their major components (the solid lipids CP and MM) at 2917 cm^−1^ and 2849–2850 cm^−1^ corresponding, respectively, to ν_a_C–H and ν_s_ C–H vibration modes of CH_2_. Other bands related to ester bonds were observed in 1733 cm^−1^ (ν C=O); 1463 cm^−1^ and 1342–1343 cm^−1^ (δ C–H in CH_2_)^15,49^. Finally, characteristic bands of P68 molecule were observed at 963 cm^−1^ to 1108 cm^−1^ and attributed to the symmetrical structure of C–O and the asymmetric stretching vibrations of C–O in the ether groups of –OCH_2_CH_2_ residues, repeated throughout the structure of P68^50^.

ATR-FTIR spectra of NLC loading TTC showed similarities to those of the control NLC spectra (without TTC), indicating that incorporation of the anesthetic did not affect the overall arrangement of the NLC excipients in the nanoparticle^8^. Among all the NLC spectra, only those of formulations containing tetracaine exhibited typical bands of pure TTC at 1278 cm^−1^ (C–N stretching) and 1683 cm^−1^ (C=O stretching) which were shifted to 1281–1285 cm^−1^ and 1603–1606 cm^−1^, respectively. Such displacements confirm the insertion of TTC into the NLC, probably due to interactions between the amine groups of TTC and available groups of NLC matrices, as described before^49^.

### Differential scanning calorimetry (DSC) analysis

**Figure 4:**
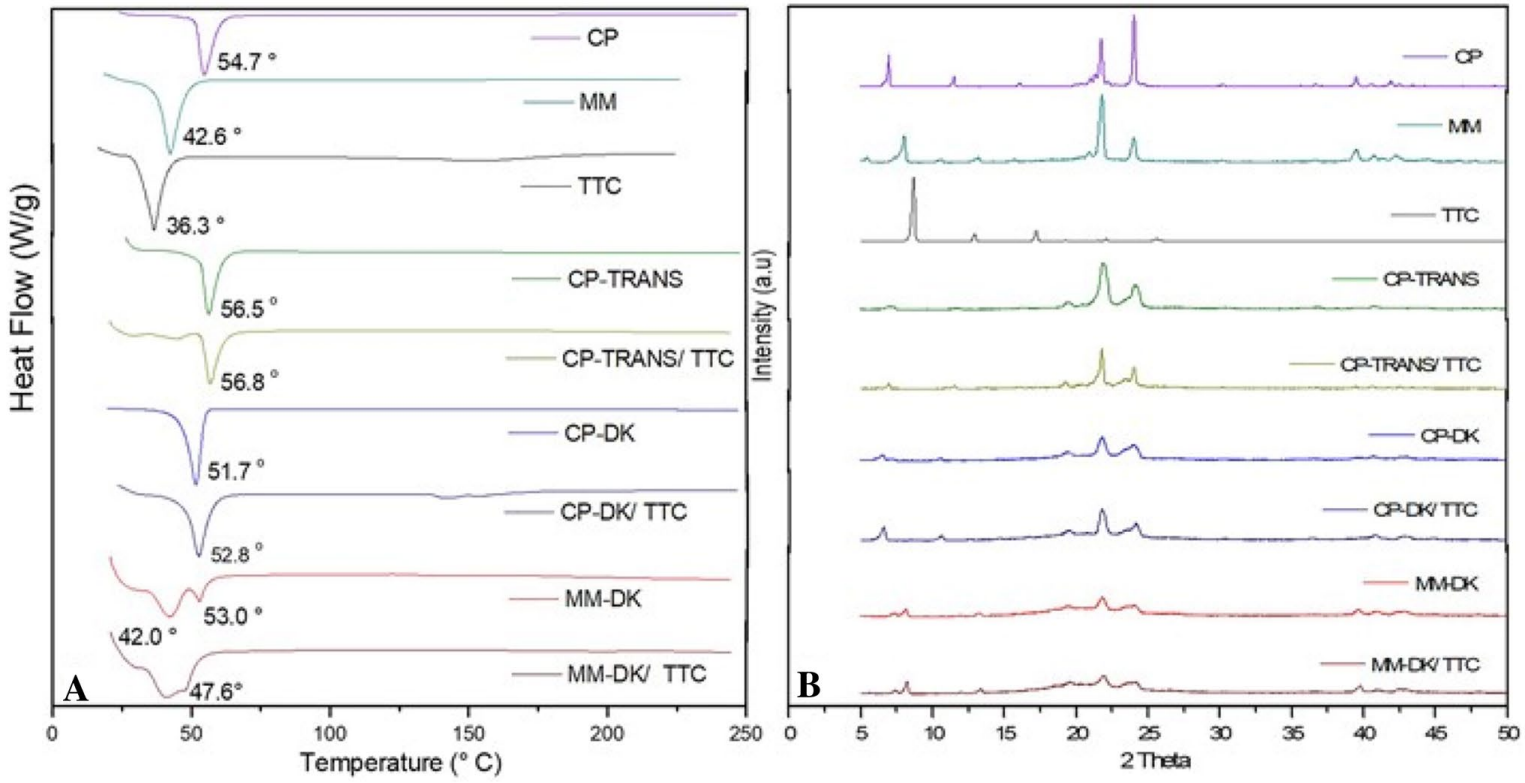
DSC (**A**) and XRD (**B**) analyses of nanostructured lipid carriers containing tetracaine (CP-TRANS/TTC, CP-DK/TTC and MM-DK/TTC), their controls (CP-TRANS, CP-DK and MM-DK), major excipients (CP, MM) and TTC base.

Figure 4A shows the thermograms obtained with the optimized NLC formulations, their major (solid lipid) excipients or TTC. The peak belonging to TTC (36.3 °C) could not be seen in any of the three optimized formulations, indicating insertion of the anesthetic inside the nanoparticles^51^. In agreement with the literature, endothermic peaks corresponding to the melting of CP and MM were observed at 54.7 and 42.6 °C, respectively^15,52^. Incorporation of the liquid lipids slightly changed the transition of cetyl palmitate to higher (CP-TRANS) or lower temperatures (CP-DK) or, in the case of myristyl myristate, to lower temperatures (MM-DK) plus the appearance of another transition at higher temperature (53 °C). The decrease in the transition of the solid lipid, observed with CP-DK and MM-DK, are expected since the liquid lipid causes a reduction in crystallinity of the solid lipid^51^. As for the shift in the transition of cetyl palmitate to higher temperatures (56.5 °C in the case of CP-TRANS) it indicates an increase in the crystallinity index of the solid lipid inside the NLC, probably because of miscibility problems with TRANS (as will be discussed latter, in the Raman imaging results). This shift was also observed in the presence of tetracaine (56.8 °C for CP-TRANS/TTC).

### X-ray diffraction (XRD) analysis

X-ray diffraction experiments provided information regarding the crystalline structure of NLC and TTC. Figure 4B shows the diffractograms of the optimized NLC formulations, their controls, major excipients (solid lipids, CP and MM) and TTC. The diffractogram of TTC showed three typical peaks at 2θ = 8.66°, 12.90° and 17.23°, and other peaks of lower intensity, confirming the crystalline nature of the anesthetic^53^. These narrow peaks were not detected in the diffractograms of the optimized formulations, indicating that the anesthetic was solubilized in the lipid matrix of the NLC. In addition, diffractograms of control NLC (CP-TRANS, CP-DK and MM-DK) were different from those of the pure solid lipids (CP and MM), showing peaks of lower intensities (2θ = 6.94°, 21.72°, 24.02° for CP, and 2θ = 7.96°, 21.78°, 23.97° for MM, respectively). These data indicate the lower crystallinity of the NLC lipid matrix in relation to the pure lipids (CP, MM), thus reflecting a less ordered structure that results from the presence of liquid lipids in the core of the nanoparticles.

The diffraction patterns of TTC-containing particles CP-DK/TTC, MM-DK/TTC and their respective controls (CP-DK and MM-DK) were similar, confirming that addition of TTC did not change the overall organization of these nanoparticles, in agreement with TEM (Fig. 2) and DSC (Fig. 4A) data. In the case of CP-TRANS/TTC sample there is a narrowing in the more intense CP peaks at 21.72° and 24.02° promoted by TTC, indicating that the anesthetic increases the crystallinity of cetyl palmitate, in agreement with DSC results.

### Small angle neutron scattering

**Figure 5:**
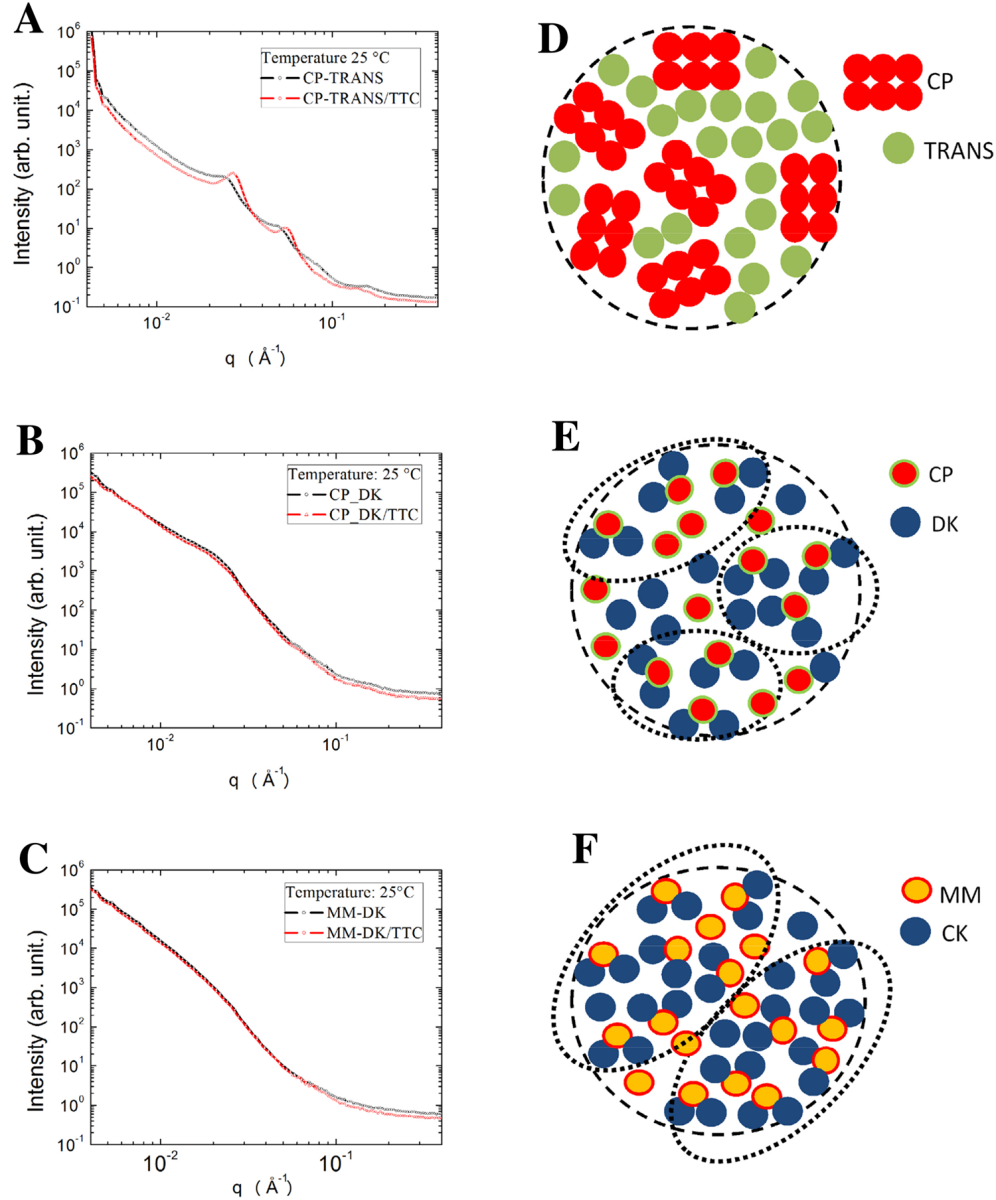
SANS data, measured at 25 °C, for the optimized NLC formulations and their controls (without TTC): (**A**) CP-TRANS, (**B**) CP-DK, (**C**) MM-DK. (**D**–**F**) Schematic representation of the lipid arrangements in the inner core of the three kind of nanoparticles (for the sake of clarify the surfactant molecules are not represented). Notice that a lamellar structure was detected in the core of CP-TRANS particles (**D**) while hydrophobic clusters (dotted lines) were observed between the lipids CP-DK (**E**) and MM-DK systems (**F**).

SANS measurements were performed in order to get further information on the structural organization of the optimized NLC, with and without tetracaine. The samples were prepared in D_2_O to reach a significant contrast between the solvent and the nanoparticles. First, all the NLC systems exhibit negligible changes when measurements were conducted at 25 °C and at 37 °C (as shown in Figure S1 for CP-DK/TTC and MM-DK/TTC). SANS data then revealed several systematic tendencies in the internal arrangement of the nanoparticles (Fig. 5). For those prepared with cetyl palmitate and Transcutol (CP-TRANS/TTC, CP-TRANS) correlation peaks in the SANS curves indicated the existence of lamellar structures inside the NLC (Fig. 5A), in agreement with previous reports in the literature, obtained with Electron Paramagnetic Resonance^45^ and molecular Dynamics^54^. Indeed, among the blends of solid and lipid lipids tested, cetyl palmitate and TRANS have the largest difference in polarity, and their SL:LL molar ratio (0.66) was the smallest among the three optimized formulations (Table S3). Because of that, the lamellar structure revealed by SANS results from the reorganization of CP molecules in the lipid NLC core, avoiding the contact with TRANS molecules (see “Discussion” below). Interestingly, and in agreement with that, the Design of Experiments study revealed that only for the CP-TRANS formulation (Fig. 1A) the amount of solid lipid (CP) played a significant effect, determining increased particles size.

Moreover, inclusion of tetracaine in the CP-TRANS nanoparticles induced a variation in the observed lamellar structure, since the 1 0-plane spacing (d10) diminished from 259 Å in CP-TRANS, to 230 Å in CP-TRANS/TTC. Such reduction in the lamellar interplanar distances (d10) suggests that tetracaine interacts with the CP molecules, decreasing the thickness or the lamellae by promoting lateral expansion, a phenomenon already observed for TTC in monolayers and bilayers^55,56^. TTC really causes dynamic rearrangements in lamellar phases, as recently demonstrated by Hu et al. in dioleylphosphatidylcholine supported bilayers, increasing the lipid chain mobility and even inducing the formation of curved tubular structures prior to membrane disruption, at high TTC:lipid ratios^57^.

No such correlation peaks were observed in the SANS profile of the NLC prepared with Dhaykol 6040 as the liquid lipid (Fig. 5B,C). But to gain a further insight about the DK-based NLC systems, we modeled the SANS data using Eq. (3), as shown in Figure S1 (“Supporting information”). The analysis of the correlation length parameter for the CP-DK and MM-DK samples showed that the latter had a bigger correlation length ($\xi_{{MM{ - }DK}} = 78.68$ Å) than the former ($\xi_{{CP{ - }DK}} = 44.69$ Å) suggesting that MM-DK nanoparticles had greater hydrophobic clusters (nanoclusters formed by the hydrophobic interactions between solid and liquid lipids)^52^, in comparison to CP-DK.

In the representations at Fig. 5D–F we depicted the different organizations proposed for the solid and liquid lipids inside the NLC, revealing the hydrophobic clusters observed for CP-DK and MM-DK, but not CP-TRANS. The size of the hydrophobic clusters did not change in the presence of TTC, nor with temperature (Figure S1).

### Physicochemical stability studies

**Figure 6:**
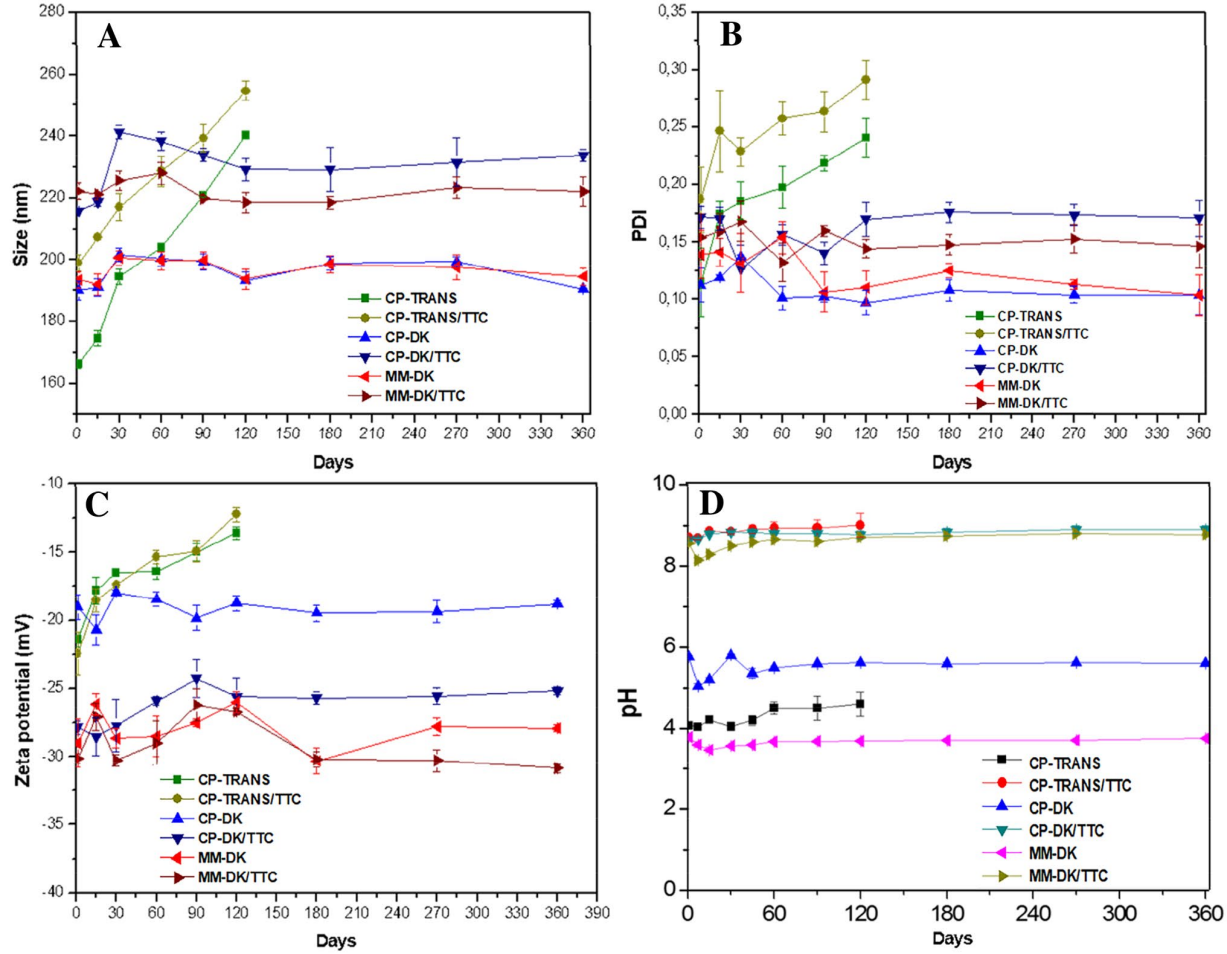
Stability of the optimized formulations during 365 days of storage at 25 °C, considering size (**A**), PDI (**B**), ZP (**C**) and pH (**D**).

A long-term stability study was conducted with the optimized NLC formulations and their controls, by monitoring particle size, PDI, ZP, pH and visual aspects such as color and homogeneity for 365 days at 25 °C. The pH of the formulations remained in the range of 8.0–8.5. CP-DK/TTC and MM-DK/TTC and their respective controls did not show any significant variation by visual inspection or in any of the analyzed parameters over time (Fig. 6), reflecting the physical stability of these formulations (i.e*.* maintenance of nanoparticles structure).

On the other hand, CP-TRANS/TTC and CP-TRANS showed a significant increase in nanoparticle size and polydispersity (254.7 nm and 0.29, respectively, for CP-TRANS/TTC after 120 days) with ZP values tending to zero (p < 0.05) during storage (Fig. 6). Visual analysis confirmed the instability of this formulation, with phase separation starting after 30 days that prevented analyses after 120 days. Therefore, CP-TRANS NLC was found unstable over time, with changes in particle size, polydispersity and ZP values compatible with particle aggregation^37,21^.

### Miscibility of lipid excipients measured by Raman mapping

In an attempt to get more information on the stability of the formulations, Raman imaging analyses were used to evaluate the miscibility of their lipid components. Figure S2 shows Raman spectra of solid and liquid lipids, and their mixtures. These spectra were very similar to prior reports in literature and also previous works from our group^16,22^. As expected, solid lipids had narrower bands than liquid lipids because of their more ordered molecular structures. Assignment of the main bands of each excipient is given in Table S4.

**Figure 7:**
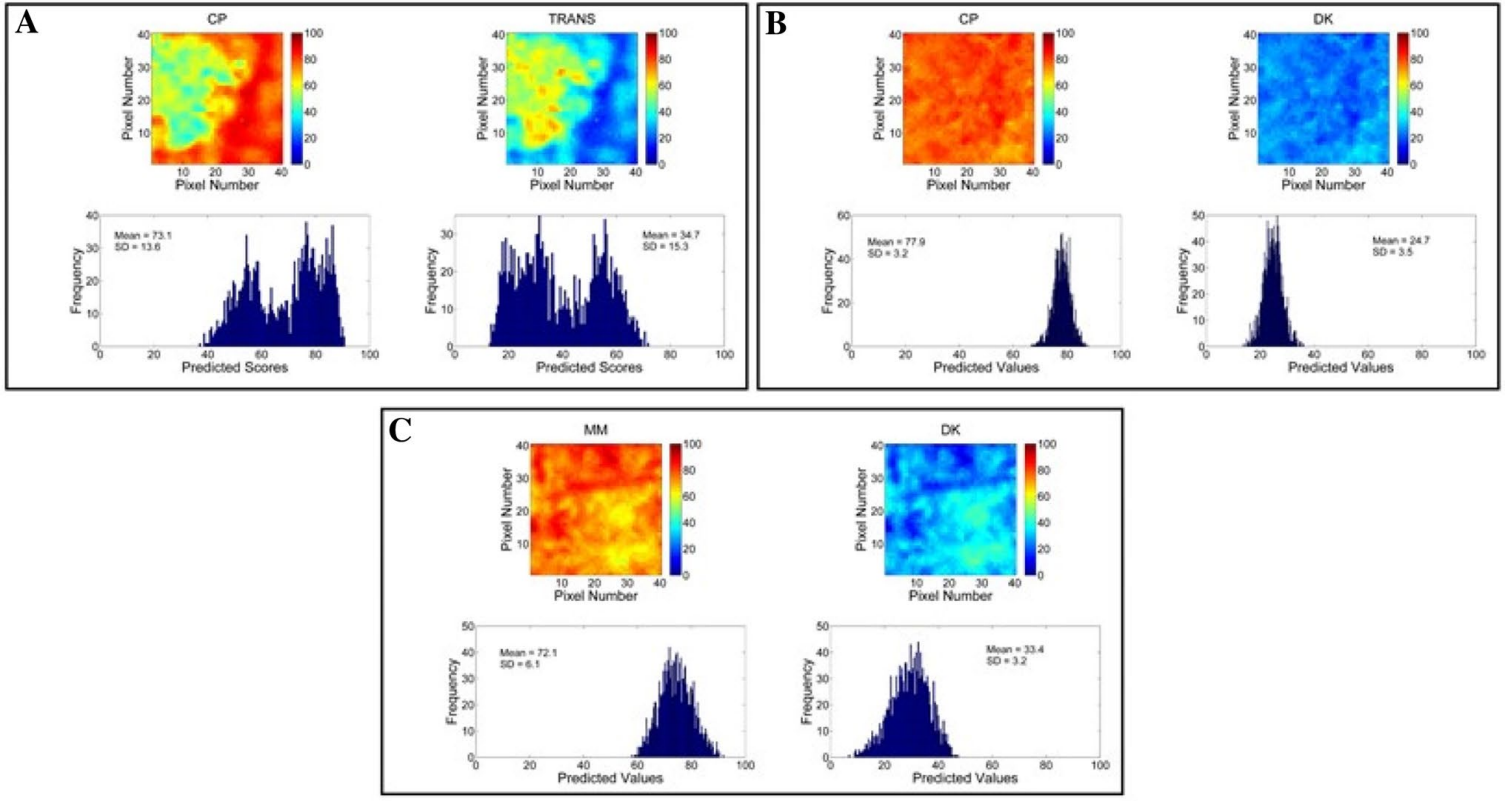
Raman images: distribution maps (top) and histograms (bottom) of predicted values for: (**A**) CP-TRANS; (**B**) CP-DK, and (**C**) MM-DK. For each sample, solid lipids (CP, MM) are represented in the left and liquid lipids (TRANS, DK) in the right.

In this case, univariate methods (i.e. single wavenumber) could not be used to treat the data due to the high spectral overlap (Figure S2). Therefore, and since the spectrum of each individual component was available, the multivariate CLS method was employed, allowing the use of all spectra information to generate the chemical images. Figure 7 shows chemical imaging and histograms obtained for the three pairs of solid and liquid lipids of the optimized NLC. Each pair of SL/LL is shown on the left and right sides, respectively of Fig. 7A–C. The predicted mean scores and their ranges for each component in the pixels are given in the histograms. The scores are in the same scale, so they can be directly compared to evaluate lipid miscibility. SD_hist_ values of 13.6, 3.2 and 6.1 were found for the lipid mixtures CP-TRANS, CP-DK and MM-DK, respectively. Pixels with 0 or 100 score values would indicate full immiscibility between excipients, but they were not observed in any of the mixtures. CP-TRANS sample showed very wide histograms, with two maxima (Fig. 7A). Such behavior is a clear indication of aggregation, with CP concentrated in the right side (red in the chemical map) and TRANS condensed in the left side (green in the chemical map). The histograms of CP-DK were the narrowest ones (Fig. 7B), and MM-DK showed an intermediate behavior (Fig. 7C). It should be noted that unlike CP-TRANS, the last two systems showed a single distribution, with only one maximum for each (SL and LL) excipient. According to the Raman image analyses, the degree of miscibility in three mixtures decreased in the order: CP-DK > MM-DK >  > CP-TRANS, corroborating the SANS results (Fig. 5). These results also explain the instability of CP-TRANS under storage, as revealed by DLS (Fig. 6), and confirm the applicability of Raman mapping for the selection of excipients in pharmaceutical studies^22,23^. So, for stability reasons, only the formulations containing DK as the liquid lipid were used in subsequent steps.

### In vitro release kinetics

**Figure 8:**
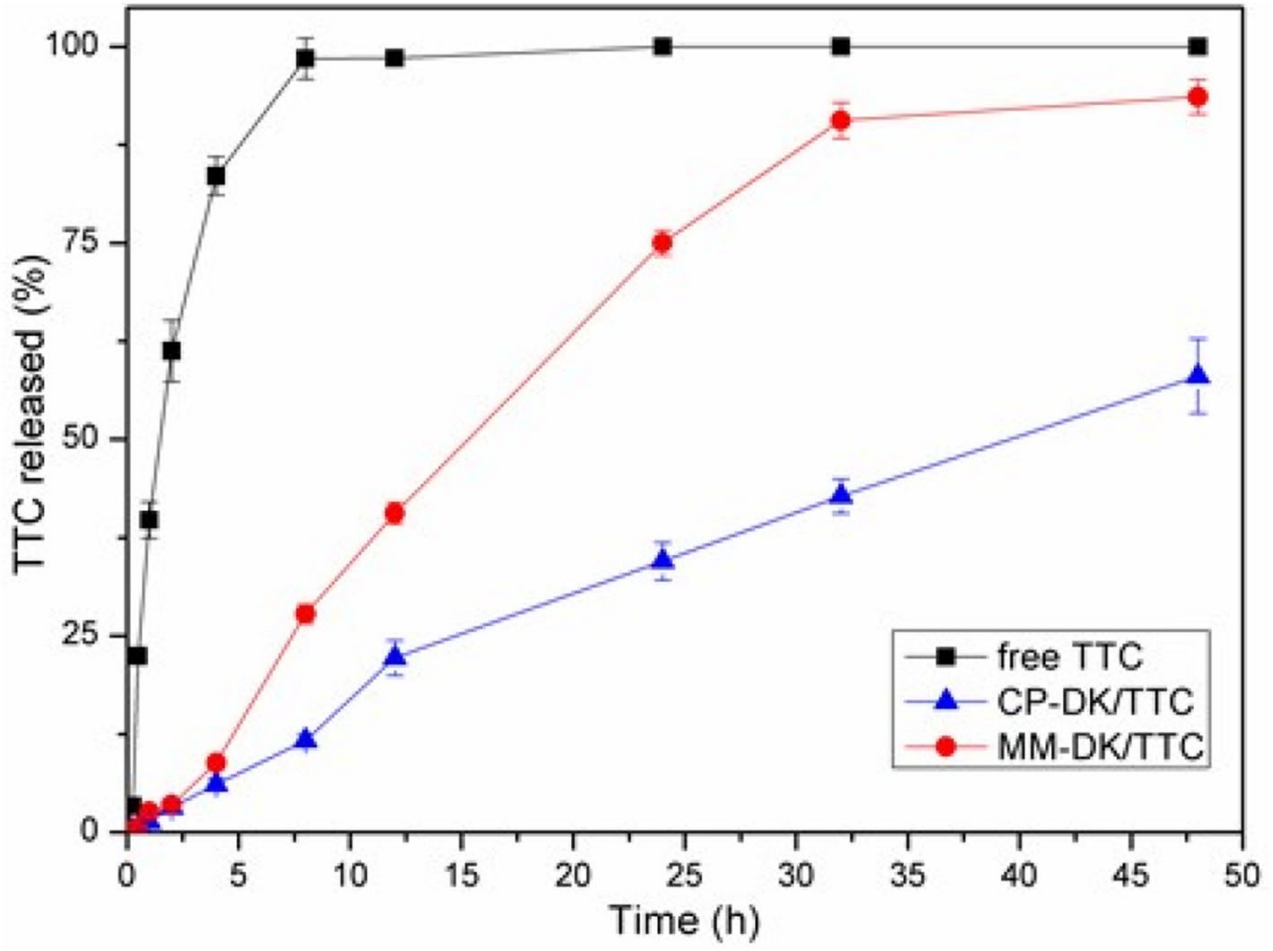
In vitro release profiles of 4% tetracaine in solution (free TTC) or encapsulated in optimized (CP-DK/TTC and MM-DK/TTC) nanoparticles, at pH 7.4 and 37 °C (n = 6).

The in vitro release of TTC, in solution and encapsulated in CP-DK/TTC and MM-DK/TTC formulation (Fig. 8), was measured during 48 h at 37 °C. TTC in solution reached equilibrium (100% release) in 6 h. The NLC formulations displayed slower TTC release: 40.9% and 93.0%, respectively for CP-DK/TTC and MM-DK/TTC, after 48 h. The release curves were treated with several kinetic models, and the better fit (r^2^ = 0.9991, and 0.9978, for CP-DK/TTC and MM-DK/TTC, respectively) was found with the Korsmeyer–Peppas model (Eq. 4). The determined values of *n* were 0.47 (CP-DK/TTC) and 0.49 (MM + DK/TTC). According to the Korsmeyer-Peppas model, *n* values from 0.43 to 1 indicate an anomalous, non-Fickian transport^30^. This means that probably two mechanisms drove the release of TTC: an initial “burst” release due to the non-encapsulated TTC (ca. 35%, see Table 2), and a sustained release regimen related to the fraction of TTC loaded by the nanoparticles. The prolonged release of TTC encapsulated in CP-DK/TTC correlates well with the higher degree of miscibility in the lipid core of these nanoparticles in comparison to MM-CK/TTC, as revealed by Raman Imaging (Fig. 7) and SANS (Fig. 5) data. This modeling confirmed the NLC ability to extend the release of local anesthetics, as previously observed^16^.

### In vitro cytotoxicity tests

**Figure 9:**
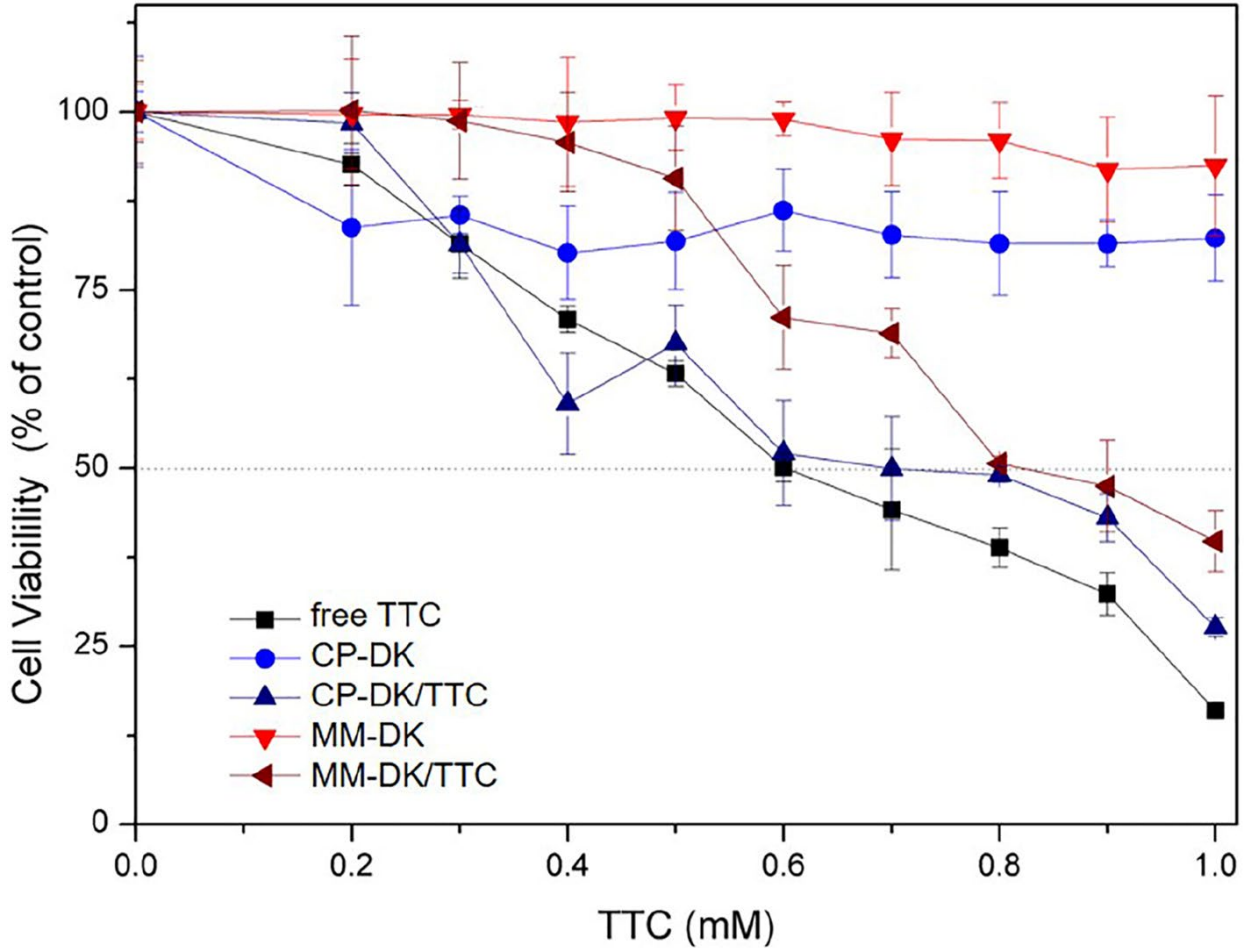
Cytotoxicity evaluation—trough the MTT test—on Balb/c 3T3 fibroblasts treated for 24 h with TTC in solution (free TTC) or encapsulated in the nanoformulations (CP-DK/TTC, MM-DK/TTC), and their controls (CP-DK, MM-DK), n = 3.

Finally, we evaluated the cytotoxicity of the optimized (CP-DK/TTC and MM-DK/TTC) formulations through the MTT test (Fig. 9), in cultures of murine Balb/c 3T3 fibroblasts. After 24 h of treatment, the IC_50_ of free TTC was 0.6 mM, in good agreement with the literature^24^. Slightly higher IC_50_ values were detected with CP-DK/TTC and MM-DK/TTC formulations (0.7 mM and 0.9 mM, respectively), showing that the NLC formulations decrease the intrinsic cytotoxicity of TTC, probably due to the sustained drug release over time. Similar observations were reported for TTC encapsulated in NLC composed of glyceryl monostearate, oleic acid and Tween 80^31^. Control formulations (CP-DK and MM-DK) were also tested, and they showed no (MM-DK) or low (CP-DK) effect over cell viability at the concentrations tested, indicating the safety of the nanocarrier systems.

## Conclusions

In this study, three NLC pre-formulations for tetracaine (CP-TRANS/TTC, CP-DK/TTC and MM-DK/TTC) were optimized by factorial design and their supramolecular structure was scrutinized with biophysical methods that detected evidence on the interaction of TTC with the lipid core of the NLC. The optimized formulations were capable of encapsulating TTC (%EE > 63%, %DL > 11%) at high doses (4%) and the nanoparticles promoted sustained release of TTC beyond 48 h, as evaluated in vitro*,* at pH 7.4 and 37 °C. But when SANS results revealed details on the lipid core of the NLC, a highly ordered (lamellar) arrangement was observed for CP-TRANS/TTC. In addition, Raman mapping analysis detected the low miscibility between the lipids CP and TRANS, explaining the instability of the CP-TRANS/TTC formulation during storage (Fig. 6), while CP-DK/TTC and MM-DK/TTC remained stable for 365 days, at 25 °C. The two remaining pre-formulations promoted sustained release and reduced the intrinsic toxicity of TTC over cultured 3T3 cells (MM-DK/TTC > CP-DK/TTC) in vitro. Therefore, the optimized nanoparticles prepared with DK show interesting properties as carriers for TTC administration, by different routes. Further in vivo studies are necessary to evaluate the therapeutic effect of the optimized pre-formulations and possible reduction of the systemic toxicity of TTC, in comparison to commercially available TTC products.

## Supplementary Information

Supplementary Information.
