# Supplementary material for: A pre-formulation study of tetracaine loaded in optimized nanostructured lipid carriers
Source: Sci Rep. 2021 Nov 2;11:21463. doi: 10.1038/s41598-021-99743-6 (PMC8563806; doi:10.1038/s41598-021-99743-6)
Supplement: Supplementary file 1 — Supplementary Information. [file 41598_2021_99743_MOESM1_ESM.docx]

**SUPPORTING INFORMATION**

**A pre-formulation study of tetracaine loaded in optimized nanostructured lipid carriers**

Simone R. Castro, Lígia N. M. Ribeiro, Márcia C. Breitkreitz, Viviane A. Guilherme, Gustavo H. Rodrigues da Silva, Hery Mitsutake, Ana C.S. Alcântara, Fabiano Yokaichiya, Margareth K.K.D. Franco, Daniel Clemens, Ben Kent, Marcelo Lancellotti, Daniele R. de Araújo and Eneida de Paula

**Table S1:** Composition of the three NLC containing tetracaine (CP-TRANS/TCC; CP-DK/TCC; MM-DK/TTC) prepared accordingly to the 2^3^ factorial design, and their corresponding experimental responses: size, PDI and ZP.

|  | **Factors** | | | **CP-TRANS/TTC** | | | **Responses**  **CP-DK/TTC** | | | **MM-DK/TTC** | | |
| --- | --- | --- | --- | --- | --- | --- | --- | --- | --- | --- | --- | --- |
| **Formulation** | P68 | TL | SL:LL | Size | PDI | ZP | Size | PDI | ZP | Size | PDI | ZP |
|  | (%w/v) | (%w/v) | (%w/w) | (nm) |  | (mV) | (nm) |  | (mV) | (nm) |  | (mV) |
| 1 | 2 | 16 | 70:30 | 278.8 | 0.252 | -28.1 | 328.0 | 0.189 | -31.1 | 310.5 | 0.245 | -34.9 |
| 2 | 5 | 16 | 70:30 | 193.1 | 0.153 | -18.8 | 202.8 | 0.117 | -24.0 | 223.6 | 0.216 | -27.8 |
| 3 | 2 | 20 | 70:30 | 278.8 | 0.214 | -33.2 | 412.1 | 0.200 | -36.9 | 414.0 | 0.325 | -38.8 |
| 4 | 5 | 20 | 70:30 | 192.2 | 0.141 | -17.8 | 204.6 | 0.141 | -23.0 | 229.6 | 0.177 | -24.8 |
| 5 | 2 | 16 | 90:10 | 332.8 | 0.243 | -32.6 | 506.3 | 0.245 | -36.9 | 453.3 | 0.271 | -40.3 |
| 6 | 5 | 16 | 90:10 | 202.2 | 0.215 | -20.4 | 258.7 | 0.147 | -23.3 | 223.3 | 0.146 | -31.4 |
| 7 | 2 | 20 | 90:10 | 345.9 | 0.212 | -32.2 | 544.0 | 0.230 | -35.1 | 434.6 | 0.275 | -40.7 |
| 8 | 5 | 20 | 90:10 | 202.5 | 0.184 | -19.5 | 302.5 | 0.169 | -25.1 | 240.0 | 0.153 | -32.4 |
| 9 | 3.5 | 18 | 80:20 | 261.6 | 0.193 | -23.2 | 282.9 | 0.151 | -28.7 | 320.5 | 0.204 | -24.9 |
| 10 | 3.5 | 18 | 80:20 | 240.4 | 0.182 | -20.7 | 270.5 | 0.159 | -29.7 | 284.0 | 0.182 | -30.2 |
| 11 | 3.5 | 18 | 80:20 | 249.0 | 0.194 | -22.5 | 300.4 | 0.162 | -28.0 | 270.1 | 0.214 | -28.3 |

**Table S2:** p-values of regression models (ANOVA) used to evaluate CP-TRANS, CP-DK and MM-DK as lipid matrixes of NLC formulations.

| **Parameter** | **Size** | | **PDI** | | **ZP** | |
| --- | --- | --- | --- | --- | --- | --- |
|  | Coefficient | p-value | Coefficient | p-value | Coefficient | p-value |
| **CP-TRANS/TTC** |  |  |  |  |  |  |
| Intercept | 252.5 | - | 0.200 | - | -24.4 |  |
| P68 (A) | -55.8 | < 0.0001 | -0.029 | 0.0002 | -6.2 | < 0.0001 |
| TL (B) | - | - | -0.014 | 0.0073 | - | - |
| SL (C) | 17.6 | 0.0002 | 0.012 | 0.0156 | - | - |
| AC | 12.7 | 0.0012 | 0.014 | 0.0062 | - | - |
| **CP-DK/TTC** |  |  |  |  |  |  |
| Intercept | 328.4 | - | 0.170 |  | -29.2 |  |
| P68 (A) | -102.7 | 0.0002 | -0.036 | 0.0002 | -5.6 | < 0.0001 |
| SL (C) | 58.0 | 0.0070 | 0.018 | 0.0117 | - | - |
| **MM-DK/TTC** |  |  |  |  |  |  |
| Intercept | 309.4 | - | 0.220 | - | -32.2 | - |
| P68 (A) | -87.0 | 0.0002 | -0.053 | 0.0008 | -4.8 | 0.0094 |

**Table S3**: Estimated number of excipients per nanoparticle, in each of the three optimized (CP-TRANS/TTC, CP-DK/TTC, MM-DK/TTC) formulations, calculated from the nanoparticle concentration and %EE. SL = solid lipid; LL = liquid lipid; TL = total lipid (SL + LL).

| **Formulation** | **SL ^a^**  **(.10^4^)** | **LL ^a^**  **(.10^4^)** | **P68 ^a^**  **(.10^4^)** | **TTC ^b^**  **(.10^4^)** | **SL:LL**  **(molar ratio)** | **TTC : TL**  **(molar ratio)** |
| --- | --- | --- | --- | --- | --- | --- |
| CP-TRANS/TTC | 230 | 350 | 4 | 81.7 | 0.66 | 0.14 |
| CP-DK/TTC | 170 | 210 | 3 | 64.4 | 0.81 | 0.17 |
| MM-DK/TTC | 260 | 250 | 5 | 82.8 | 1.04 | 0.16 |

^a^ Calculated from the molar concentration of each excipient divided by the number of particles in suspension, measured by NTA, according to Guilherme et al., 2019. ^b^ Calculated from the number of particles in suspension, and encapsulation efficiency (Table 2).

**Table S4:** Assignment of the Raman main bands observed in the excipients of NLC formulations.

| Raman shift (cm^-1^) | Functional group | Excipient | Reference |
| --- | --- | --- | --- |
| 2732 – 2728 | -CH | CP, DK, MM | (Anantachaisilp et al., 2010) |
| 1744 | ν -C=O | DK | (Patnaik, 2004) |
| 1440 – 1450 | -CH_2_ and -C=O in ester | CP, DK, MM | (Patnaik, 2004) |
| 800 – 900 | -CH_3_ rocking | CP, TRANS, DK, MM | (Patnaik, 2004) |
| 890 – 830 | R–O–R, in aliphatic acyclic compounds | TRANS, DK | (Patnaik, 2004) |

**
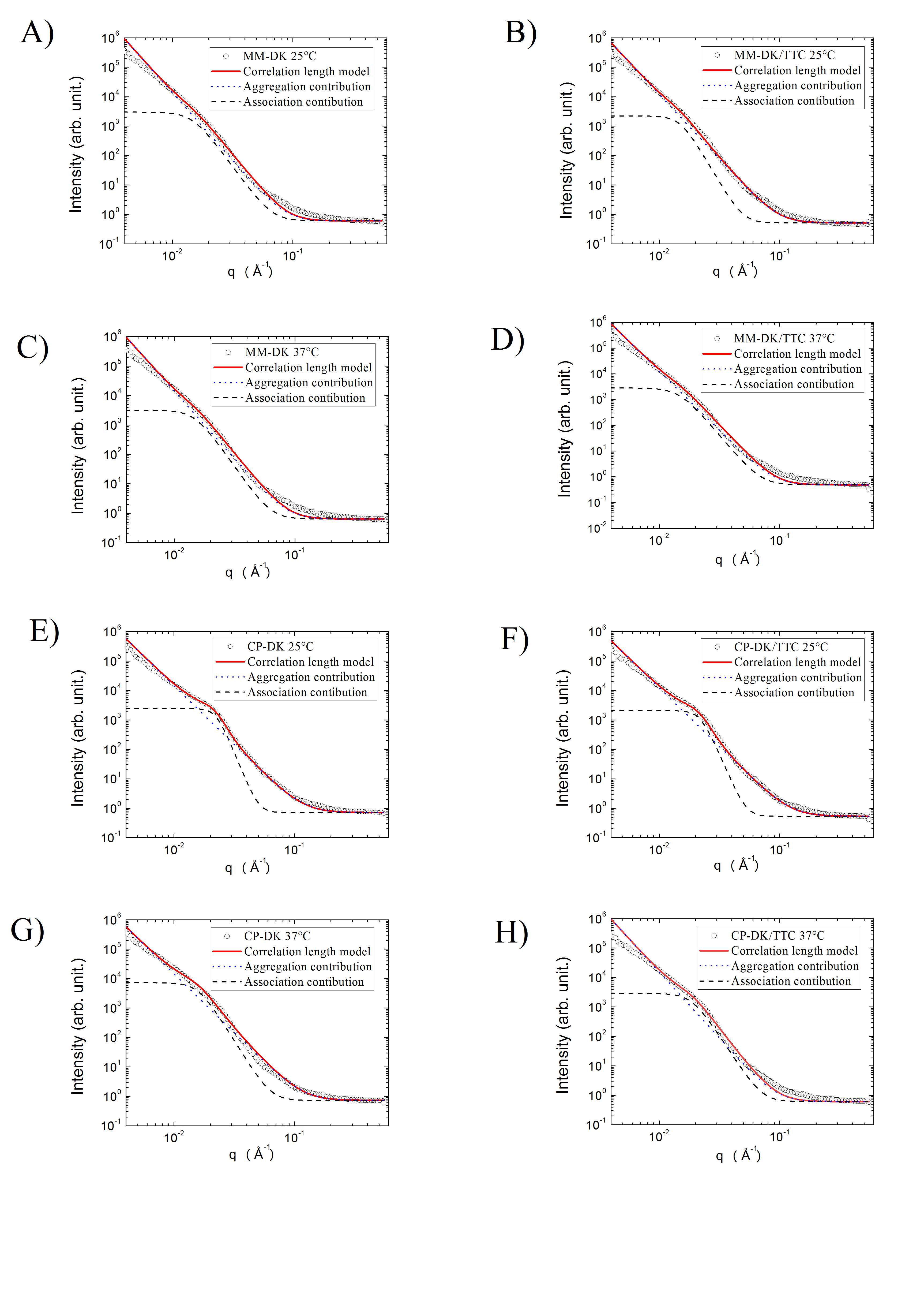
**

**Figure S1:** SANS data for: MM-DK (A) and MM-DK/TTC (B) at 25°C; MM-DK (C) and MM-DK/TTC (D) at 37°C; CP-DK (E) and CP-DK/TTC (F) at 25°C; CP-DK (G) and CP-DK/TTC (H) at 37°C. The solid lines through each curve are fits from equation 3. Dashed lines represent individual contributions due to aggregates (aggregation contribution) and hydrophobic interactions.

**
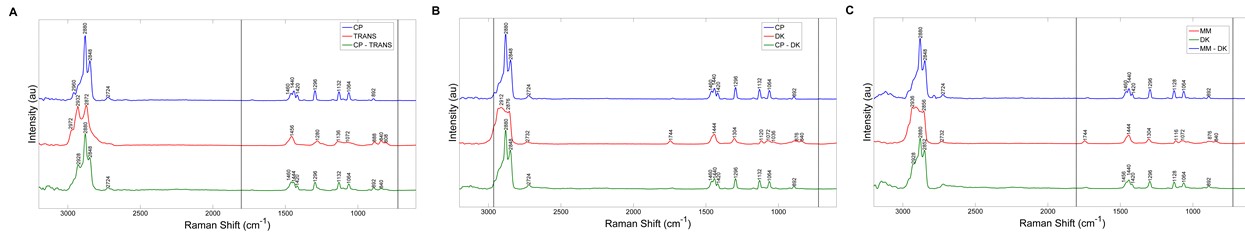
**

**Figure S2.** Raman spectra of the solid and liquid lipids of the optimized NLC, and their mixtures: (A) CP-TRANS; (B) CP-DK; (C) MM-DK. Solid lipid excipient spectra (blue, top); liquid lipid spectra (red, medium) and mean spectra of their mixtures (green, bottom).
